# A before-after implementation trial of smoking cessation guidelines in hospitalized veterans

**DOI:** 10.1186/1748-5908-4-58

**Published:** 2009-09-10

**Authors:** David Katz, Mark Vander Weg, Steve Fu, Allan Prochazka, Kathleen Grant, Lynne Buchanan, David Tinkelman, Heather Schacht Reisinger, John Brooks, Stephen L Hillis, Anne Joseph, Marita Titler

**Affiliations:** 1 Department of Medicine, University of Iowa Carver College of Medicine, University of Iowa, Iowa City, IA, USA; 2 Center for Research in the Implementation of Innovative Strategies in Practice, Iowa City VA Medical Center, University of Iowa, Iowa City, IA, USA; 3 Center for Chronic Disease and Outcomes Research, Minneapolis VA Medical Center, Minneapolis, MN, USA; 4 Department of Medicine, VA Eastern Colorado Health Care System, Denver, CO, USA; 5 Department of Psychiatry, VA Nebraska-Western Iowa Health Care System, Omaha, NE, USA; 6 The College of Nursing, University of Nebraska, Omaha, NE, USA; 7 Health Initiatives Program, National Jewish Health, Denver, CO, USA; 8 College of Pharmacy, University of Iowa Carver College of Medicine, University of Iowa, Iowa City, IA, USA; 9 College of Nursing, University of Iowa Carver College of Medicine, University of Iowa, Iowa City, IA, USA; 10 Department of Medicine, University of Minnesota, Minneapolis, MN, USA

## Abstract

**Background:**

Although most hospitalized smokers receive some form of cessation counseling during hospitalization, few receive outpatient cessation counseling and/or pharmacotherapy following discharge, which are key factors associated with long-term cessation. US Department of Veterans Affairs (VA) hospitals are challenged to find resources to implement and maintain the kind of high intensity cessation programs that have been shown to be effective in research studies. Few studies have applied the Chronic Care Model (CCM) to improve inpatient smoking cessation.

**Specific objectives:**

The primary objective of this protocol is to determine the effect of a nurse-initiated intervention, which couples low-intensity inpatient counseling with sustained proactive telephone counseling, on smoking abstinence in hospitalized patients. Key secondary aims are to determine the impact of the intervention on staff nurses' attitudes toward providing smoking cessation counseling; to identify barriers and facilitators to implementation of smoking cessation guidelines in VA hospitals; and to determine the short-term cost-effectiveness of implementing the intervention.

**Design:**

Pre-post study design in four VA hospitals

**Participants:**

Hospitalized patients, aged 18 or older, who smoke at least one cigarette per day.

**Intervention:**

The intervention will include: nurse training in delivery of bedside cessation counseling, electronic medical record tools (to streamline nursing assessment and documentation, to facilitate prescription of pharmacotherapy), computerized referral of motivated inpatients for proactive telephone counseling, and use of internal nursing facilitators to provide coaching to staff nurses practicing in non-critical care inpatient units.

**Outcomes:**

The primary endpoint is seven-day point prevalence abstinence at six months following hospital admission and prolonged abstinence after a one-month grace period. To compare abstinence rates during the intervention and baseline periods, we will use random effects logistic regression models, which take the clustered nature of the data within nurses and hospitals into account. We will assess attitudes of staff nurses toward cessation counseling by questionnaire and will identify barriers and facilitators to implementation by using clinician focus groups. To determine the short-term incremental cost per quitter from the perspective of the VA health care system, we will calculate cessation-related costs incurred during the initial hospitalization and six-month follow-up period.

**Trial number:**

NCT00816036

## Background

Smoking remains the leading preventable cause of death in the US, accounting for approximately one of every five deaths (420,000 people) each year [1]. The burden of tobacco-related illnesses in the US Veterans Administration (VA) population is particularly high, and the prevalence of smoking is estimated to be 21 to 40% higher in veterans than in the general population [2,3]. Hospitalization has been identified as a 'teachable moment' for many smokers [4]. Nearly all VA hospitals have become smoke-free, and can provide a supportive environment in which smokers are not exposed to their usual external cues to smoke [5]. Moreover, approximately 50% of hospitalized smokers are ready to quit within 30 days [6], and 80% are willing to discuss smoking cessation with a counselor during hospitalization [7]. Data from the External Peer Review Program (a contracted review of the quality of VA care) suggest that over 90% of VA smokers receive advice to stop smoking during hospitalization; however, the quality and scope of such counseling is unclear.

Although adherence to smoking cessation guidelines has been actively promoted since 1997, VA hospitals typically do not facilitate cessation interventions in hospitalized smokers by providing inpatient staff with appropriate education, resources, and performance feedback, as recommended by US Public Health Service (USPHS) guidelines [1]. Additional institutional barriers in the VA include: a specialty focus on smoking cessation counseling in the majority of VA hospitals, in which most patients are referred to a multi-session smoking cessation program [8,9]; lack of continuity of care after discharge, with limited opportunities to promote continued abstinence [5]; and variable policies and practices in the dispensing of drug therapy for cessation that may contribute to the under use of effective pharmacotherapy [10]. With regard to referral, few hospitalized smokers attend smoking cessation classes or clinics after discharge [11]. In the VA, the primary reasons that patients do not follow up are: access problems (36%), lack of commitment to quitting (35%), and unhappiness with the group format (14%), which is the primary counseling format offered in many VA hospitals [12]. In addition, the delay between referral and an initial smoking cessation appointment can be critical [8], as a large proportion of smokers relapse within one week of quitting [13,14].

VA hospitals are challenged to find resources to implement the types of programs shown to be effective in research studies. Effective cessation programs typically include a high-intensity inpatient component (≤ 1 hour of face-to-face counseling) combined with sustained relapse prevention measures (≥ 4 weeks of counseling) and pharmacotherapy [11,15-17]. Much less is known about the effectiveness of 'hybrid' interventions that combine low-intensity inpatient counseling (defined as a single session lasting 10 minutes or less)[1] with sustained relapse prevention delivered by non-research personnel. Such strategies may be more realistic in practice than high-intensity interventions, as they place fewer demands on inpatient staff and are consistent with the 'ask, advise, and refer' model of cessation counseling that has been promoted in primary care [18].

### Study aims

The proposed study will test an implementation intervention to increase quit rates in hospitalized smokers in noncritical care settings by facilitating staff nurses' delivery of recommended smoking cessation services and reducing patient barriers to participation in cessation counseling. Of all the members of the inpatient team, VA staff nurses (including registered nurses (RNs) and licensed practical nurses (LPNs)) are best positioned to deliver a brief smoking cessation intervention because of their ready access to patients and education in patient education and counseling. Several controlled trials have demonstrated that nurse-delivered counseling can increase quit rates in hospitalized patients [19-22]. Although most of these trials have employed high-intensity interventions [11], low intensity counseling by staff nurses can also yield favorable results [23].

Thus, the primary aim of this practical clinical trial is to:

1. Determine the effect of a nurse-initiated intervention, which couples low intensity inpatient counseling with sustained proactive telephone counseling, on smoking abstinence in hospitalized patients.

Hypothesis 1a: Smoking cessation rates at three and six month follow-up, as measured by seven-day point prevalence abstinence (PPA) will be greater for intervention patients than usual care patients.

Hypothesis 1b: Intervention patients will be more likely to receive prescriptions for recommended pharmacotherapy for smoking cessation and referral to telephone counseling, compared to usual care patients.

To gain insight into mechanisms promoting the adoption of recommended practices by nurses and to determine the relative economic value of the intervention, key secondary aims are to:

1. Determine the impact of the intervention on nurses' attitudes toward and self-efficacy for providing smoking cessation counseling.

Hypothesis 2: Nurses attitudes toward cessation counseling and self-efficacy in providing such counseling will increase after being exposed to the intervention.

2. Identify barriers and facilitators to implementation of smoking cessation guidelines in VA hospitals and learn how to tailor the intervention to specific sites.

3. Determine the short-term cost-effectiveness of this implementation intervention.

## Methods

### Study design

**Figure 1:**
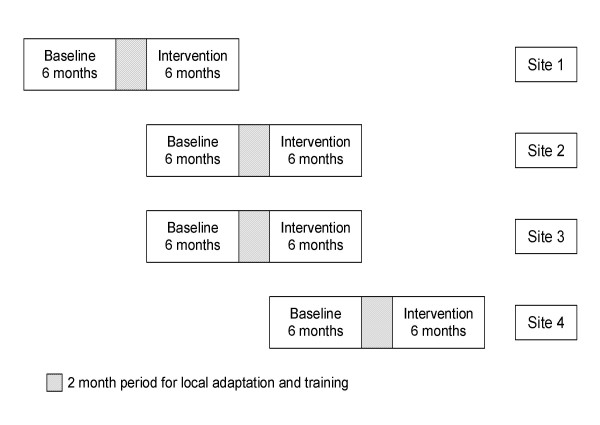
**Schematic of Study Design**.

This study is a before-after trial (with each site serving as its own control) in smokers who are hospitalized on the medical wards of four VA hospitals (Figure 1). After a six-month baseline period of enrollment, the research team will conduct focus groups of clinical staff at each site, adapt the strategy for guideline implementation, and train clinical staff at each site. A new cohort of study patients will be enrolled over the subsequent six-month intervention period. Staggering the timing of intervention across sites will allow us to collect concurrent control data for three of the four study sites during intervention; this will enable detection of system-wide trends in the delivery of cessation counseling, measurement reactivity, and other potential confounding factors within each period (except the final period)[24,25]. We considered cluster randomization by hospital, but the cost of conducting such a trial was not feasible due to budgetary constraints (as at least four sites per comparison group are typically required) [26].

### Study sites

We have selected four VA hospitals in the upper Midwest and Rocky Mountain region for this trial based on the following criteria: at least 150 general medical admissions monthly (to support recruitment targets), and support from nursing, pharmacy, and information technology (IT) leaders for this project. The variability in delivery of smoking cessation counseling across these study sites will enhance external validity, and may provide insight into the intervention's effectiveness under different baseline conditions (Table 1).

**Table 1:** Description of VA study hospitals

	**Hospital A**	**Hospital B**	**Hospital C**	**Hospital D**
Patient characteristics				
Age, mean	62.9	65.1	62.7	60.4

Gender, % male	97	96	95	94

Race, % white	97	80	85	86

Income ($), mean	21,349	26,287	20,500	19,414

General medical wards				
Annual number of admissions, n	2,988	4,388	2,701	2,806

Total number of nursing staff	42	72	60	52

% Registered nurses (RN)*	67	68	73	58

Average daily census (across wards), n	29	62	33	42

Average nursing hours per patient day	6.2	7.13	8.31	7.34

Smoking cessation services**				
Responsible division	Mental health	Mental health	Mental health	Primary care

No. of consults per month	50	60	65	30

How often do new patients start program?	1/month	1/week	NS	1/week

No. of individual counseling sessions per typical course of therapy	3	NS	2	4

Program includes group counseling	Y	Y	N	Y

Can patients receive pharmacotherapy without enrollment in smoking cessation program?	Y	Y	N	Y

Any use of telemedicine to provide cessation therapy?	Y	N	Y	N

*Source: VHA Support Service Center.  (accessed 6/13/07). Nurse staffing was estimated from local data.

**Source: Office of the Assistant Deputy Undersecretary for Health for Policy and Planning. Veterans Health Administration. Smoking and Tobacco Use Cessation Report 2005.  (last accessed 1 December 2006).

### Screening and recruitment

A study site research assistant (RA) who is not associated with the intervention will screen all medical admissions to determine eligibility by medical record review. The study sample will include general medical inpatients, aged 18 or older, who smoke at least one cigarette per day on average, regardless of their willingness to quit smoking. Current smokers transferred from intensive care units (or other monitored beds) to a general medical ward will be eligible. Exclusion criteria include: hospitalization for less than 24 hours (*e.g*., patients admitted for overnight observation); acute medical decompensation (*e.g*., acute respiratory failure requiring intubation, cardiac arrest, septic shock); altered mental status; unstable psychiatric disorder (*e.g*., acute psychosis); dementia; communication barrier (unable to speak English, hard of hearing, aphasic); pregnancy; and terminal illness (<12 month life expectancy). No patient will be included unless they provide informed consent and agree to be contacted by telephone during follow-up.

### Intervention: Application of the Chronic Care Model (CCM) in smoking cessation

The VA/Department of Defense clinical practice guideline for the management of tobacco use recommends that hospitalized patients should have smoking status documented in the medical record, should be advised to quit, should receive smoking cessation medication and counseling, and should be referred for continuing support upon discharge [27]. The challenges in designing an effective inpatient smoking cessation program are to find efficient strategies that empower clinicians to capitalize on opportunities for counseling inherent in the hospital setting and arrange for comprehensive smoking cessation therapy that extends beyond the hospital stay. In addition, many hospitalized smokers have reduced capacity to engage in discussions about smoking cessation because of medical instability, altered mental status, and the psychological stresses of acute illness [6]. Given the relapsing and remitting course of tobacco dependence, the CCM provides a framework for addressing these challenges and for improving patient outcomes [28-30], as discussed below.

### Practice redesign and nurse training

**Figure 2:**
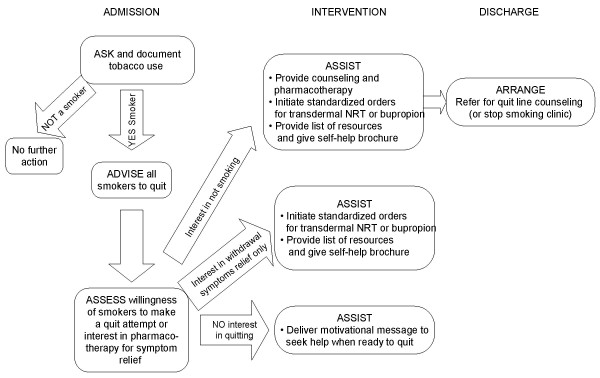
**Algorithm for Treating Tobacco Use and Dependence in Hospitals**.

For nurses, the delivery of cessation guidelines is potentially influenced by practice-related factors, including the perceived ability to offer advice (*e.g*., time pressure, urgencies of acute care, cessation skills) [31,32], perceived support of clinical leadership [33], perceived autonomy [34], and attitudes toward cessation counseling. In one survey of 369 general practice nurses, 65% believed that advice from a nurse to quit smoking was ineffective [35]. Like physicians, nurses may not perceive smoking cessation to be a high priority in clinical care or believe that they have the time to perform recommended preventive services, particularly with pressures to reduce length of stay. Our training strategy will seek to: improve staff nurses' attitudes toward the delivery of smoking cessation interventions; improve nurses' self-efficacy by providing an opportunity to practice cessation counseling and critical thinking skills with colleagues (using role-play, case studies, and clinical simulations); and use peer leaders to provide informal coaching and feedback. During an initial 30-minute, small group training session, members of the research team will present baseline performance data on cessation measures, demonstrate use of a computerized nursing reminder and referral order for telephone counseling in the VA computerized patient record system (CPRS), and demonstrate principles of cessation counseling [36,37]. Specifically, nurses will be trained on how to assist patients in moving toward change and how to recognize and manage resistance to behavior change [38,39]. Nurses will also receive a pocket card showing the smoking cessation algorithm (Figure 2) [40], standardized messages to encourage quitting, and a list of strategies for more challenging situations [23]. To supplement face-to-face training, we will also develop an online tutorial that includes case studies, video clips showing effective stage-based counseling, and tips on using computerized tools (nursing clinical reminder, quit line referral). In practice, clinicians are generally more motivated to learn if the training develops skills for solving practical problems and appeals to the clinician's sense of professional identity (*e.g*., 'as a nurse, this is something that I should do in my practice')[41].

### Self-management support

Patient self-management support will consist of brief inpatient counseling, pharmacotherapy, and follow-up telephone counseling after discharge. Because of its convenience, telephone counseling has emerged as a popular and effective alternative for delivery of self-management support [42-45]. Moreover, the importance of sustained relapse prevention has been demonstrated in systematic reviews to be an essential component of smoking cessation [11,17,46]. In a recent randomized controlled trial (RCT) of comprehensive telephone care versus routine care in outpatient VA smokers, telephone care resulted in a higher rate of six-month prolonged smoking abstinence at 12-month follow-up (13 versus 4%, p < 0.001) and 30-day abstinence from smoking (19% versus 13%, p = 0.028) [47]. The linkage of patients to community resources such as tobacco quit lines has also been advocated as a sustainable strategy for providing in-depth cessation counseling in busy practice settings [29].

The goals of brief inpatient counseling are to assess patients' willingness to quit, motivate patients to abstain from smoking during and after hospitalization, and assist smokers in formulating a plan for continued abstinence after discharge within five to ten minutes. At admission (or as soon as the patient's acute medical condition has stabilized), staff nurses will be trained to perform the 'five A's' of smoking cessation counseling (ask, advise, assess, assist, and arrange follow-up) [48], to assist patients in moving toward change, and to recognize and manage resistance to behavior change [38,39]. Staff nurses will also be instructed in how to personalize the counseling for patients' admission diagnoses and smoking-related comorbidities, as smokers are more likely to quit if they perceive that they are susceptible to the harmful effects of smoking and perceive greater benefits of quitting) [49,50]. In addition, staff nurses will assess interest in receiving pharmacotherapy to help relieve nicotine withdrawal symptoms.

Patients who express an interest in quitting will be shown a 12-minute educational program on closed-circuit television, produced specifically for hospitalized VA smokers, and will receive a self-help guide to smoking cessation ('Clearing the Air: Quit Smoking Today', developed by the National Cancer Institute) [51]. Similar educational aids have been effectively used as an adjunct in counseling hospitalized smokers [7,20], and can reduce the time required for bedside counseling.

Nurses will be trained to electronically refer those patients who are willing and ready to make a quit attempt for proactive telephone counseling using a computerized referral process, based on evidence that VA patients who receive such counseling are more likely to quit [47]. Within 48 to 72 hours of discharge, a quit line counselor will call to confirm the patient's interest in quitting, congratulate the patient on his/her decision to stop smoking, and provide encouragement to reinforce self-efficacy. For those patients who are ambivalent about quitting, the counselor will focus on increasing motivation to quit. For those patients who remain committed to quitting, the counselor will focus on relapse prevention [52].

Follow-up calls will be made using a relapse-sensitive schedule (rather than at equal intervals) [13], with flexibility to accommodate the patient's needs (up to seven calls over three months) [47,53]. Telephone counselors will document the initial and follow-up contacts in CPRS using progress note templates for smoking cessation [54]. A final report summarizing events pertinent to the quit attempt will also be entered into CPRS shortly after the final counseling session (and will be mailed to the patient's non-VA primary care clinician, if applicable). Enabling bi-directional communication with primary care will integrate the telephone counseling into the patient's ongoing care [55,56]. We will also offer one additional course of quit line counseling for relapsed smokers (*i.e*., those who fail in their initial quit attempt).

### Clinical information system

Presenting clinical reminders in a clear, simple format and coupling them with immediately actionable items is essential to enhance their effectiveness [57,58]. Streamlining the presentation of reminders and integrating them into the clinician's work flow is particularly important for nurses, who typically move rapidly from patient to patient, have little time to work through complex algorithms, and need information to be readily available [59]. The study team will work with nurse managers and CPRS clinical application specialists at each site to adapt the nursing intake form to reflect the five A's with the end-user in mind [60].

The smoking cessation template in CPRS will be designed to allow staff nurses to select and print patient self-help materials and to generate a consult for telephone care counseling. 'Quick orders' for pharmacotherapy will include recommended dosages for NRT, bupropion, and second-line therapies (and a list of contraindications to their use). The telephone care referral form will be adapted from 'fax to quit' forms that have been used successfully in primary care. System changes of this type can increase clinicians' ability to help patients quit smoking and their motivation to provide cessation counseling [18]. The research team at each site will query CPRS every two months to determine the proportion of smokers given assistance in quitting (referral for telephone counseling ordered, pharmacotherapy ordered), and these process measures will be presented to staff nurses during the intervention period.

### Organizational change

In the two months between the baseline and intervention periods, we will conduct a focus group that includes one or two of each of the following staff at each site: staff nurse, nurse manager, resident, attending physician, pharmacy director, and smoking cessation counselor (six to nine members in total). The focus groups will ask participants to: describe local smoking cessation practices and the 'smoking culture,' identify barriers and facilitators to changing smoking cessation practices, and suggest strategies that will facilitate change at the facility. Focus groups will also allow the study team to examine work place culture, to understand group norms, and to elicit local perspectives that may deviate from (or challenge) conventional beliefs [61,62]. Summaries of the focus groups will be provided to peer leaders (see below) and will be used to adapt the strategy for guideline implementation to the local context [63-65].

With input from the director of nursing and nurse managers at each site, we will identify a peer leader on each medical ward with the attributes of a change champion [59] and the ability to model counseling skills (five A's), troubleshoot issues during implementation, and assist in presenting group feedback to nursing staff [40]. Peer leaders will receive additional training in smoking cessation counseling and in monitoring the practices of other staff nurses by direct observation and medical record audit (using spot checks). By influencing colleagues through small group discussions, informal consultations, and feedback, the peer leader can facilitate adherence to guidelines [66]. A nurse investigator on the study team will contact each peer leader monthly during the intervention to provide ongoing support and to identify and resolve barriers to change. Notes on these conversations will also be documented in the study log for each site. In one primary care trial, the presence of a clinic champion was strongly associated with referral of smokers to a quit line (OR = 3.4, 95% CI = 2.4-5.0)[67], and local champions have emerged as key determinants of organizational innovation [59,68,69].

### Usual care

During the baseline period, nursing staff will be provided with general information on the rationale for the current study, but will not receive any specific training or additional resources for implementing the VA/Department of Defense Clinical Practice Guideline in inpatient medical units.

### Data collection

During both the baseline and intervention periods, patient data will be collected at four points in time: at enrollment, just prior to discharge, and at three- and six-month follow-up. Nursing staff will be surveyed just before and after implementation of the study intervention. To understand differences in performance of recommended actions across sites, we will track the fidelity with which clinician-focused elements of the intervention are implemented (*e.g*., attendance of staff nurses at training and feedback sessions, activity of peer leaders on each ward). Similarly, to understand differences in quit rates across sites, we will collect data regarding those elements of the intervention that are implemented for each study patient (*e.g*., delivery of bedside counseling, number of doses of pharmacotherapy provided, and number of telephone counseling sessions provided to patients referred to the quit line). With regard to the latter, we will also estimate the quit line connection rate by cross-referencing CPRS referrals of study patients for telephone counseling with actual quit line data [70].

### Patient-level data

After obtaining informed consent, the RA will perform a structured interview in current smokers to obtain more detailed information on smoking history (pack-years, number of quit attempts more than one day), other tobacco use (*e.g*., spit tobacco), alcohol use, overall health status, smoking-related medical comorbidities, readiness to quit smoking [23], level of tobacco addiction [71], perceived likelihood of staying off cigarettes after hospital discharge [72], and social support for quitting [6].

Just prior to discharge, the RA will ask eligible smokers whether they abstained from cigarettes during hospitalization, and whether or not the hospital staff had performed recommended smoking cessation counseling during the hospital stay. Patients will also be asked to specify whether they had received these services from their nurse, physician, or other clinician (*e.g*., respiratory therapist) [73]. Patient recall of advice to quit smoking has been shown to be reasonably accurate in assessing performance of guideline-recommended actions in clinical practice (sensitivity 92%, specificity 82% for advice to quit) [74].

At three- and six-month follow-up, research interviewers will contact patients by telephone about their smoking over seven days and 30 days prior to the interview (seven-day and 30-day, PPA), any quit attempts (>24 hours) since hospital discharge, and stage of change. At each follow-up, we will also determine prolonged abstinence (after a one-month grace period). All follow-up interviewers will be blinded to treatment assignment. Patients who report abstinence at six-month follow-up will be mailed a collection kit for salivary cotinine determination (with a follow-up telephone reminder to return the sample). A cutoff of 20 ng/ml will be used to determine abstinence, as this threshold is associated with high sensitivity and specificity (>90%) [75]. To calculate the biochemically confirmed six-month quit rate, we will adjust the seven-day PPA for the results of the cotinine analysis.

### Nurse-level data

Pre- and post-intervention attitudes toward smoking cessation counseling will be measured by a decisional balance questionnaire that includes 10 items that reflect positive attitudes and 10 items that reflect negative attitudes toward the delivery of smoking cessation assistance [76]; in addition, we will also ask staff nurses about self-efficacy and role satisfaction in helping patients stop smoking [77]. As nursing performance can also be influenced by job satisfaction and work environment [78], the survey will also assess perceptions of professional status (using a seven-item subscale of the Index of Work Satisfaction [79]) and staffing and resource adequacy (using a four-item subscale of the Practice Environment Scale [80].

### Summative evaluation of the intervention

At the end of the intervention period, we will conduct semi-structured telephone interviews with approximately 32 patients. Within each site, we will randomly sample two intervention patients from each of four stage of change groups. Sampling for interviews will continue until data are saturated (*i.e*., when no new data are forthcoming in interviews within each site) [81]. The interviewer will initially ask the patient to describe his/her experiences with smoking and any conversations about smoking with hospital staff during the index hospitalization. Patients will then be asked to identify aspects of their interactions with the inpatient team that were most helpful and most difficult with regard to smoking cessation. Patients will also be asked about the transition to outpatient care and any attempts to maintain abstinence following hospital discharge. New questions will be added, and the original questions will be refined to capture issues emerging from the data as the interviews progress.

In order to evaluate the acceptability of the intervention to nursing staff and its likelihood of being maintained, we will conduct semi-structured, one-on-one interviews of one peer leader and a random sample of five staff nurses from each site (or until saturation is reached). The questions are designed to elicit stories from the staff nurses in an effort to capture how the intervention was implemented in practice [82]. In addition, they will be asked about the usefulness of the intervention, their future plans for using the intervention (or parts of it), and their suggestions for further refinements. All telephone interviews will be tape recorded, transcribed, and reviewed by the interviewer for accuracy.

### Sample size calculations

Based on the volume of admissions at each study hospital, we plan to screen 7,548 potential subjects who are hospitalized on medical wards for greater than 24 hours during the enrollment period at the four sites. Based on chart review, we estimated smoking prevalence at each site (weighted average = 23%, range 17 to 32%), and determined that approximately 80% of identified smokers will be eligible to participate in the proposed trial. Assuming that 84% of eligible patients consent to participate (based on a recent VA smoking cessation study [47]), we would expect 1,000 subjects to enroll in the study (500 baseline, 500 intervention). Pooling the control group data from studies of general medical inpatients, we estimate a 12% quit rate at six-months (seven-day PPA). Thus, a study sample of 1,000 patients will have 83% power to detect a seven percentage-point difference between periods in six-month quit rates. This effect estimate is consistent with that observed in prior hospital intervention trials that employed a sustained relapse prevention component [16,20,21]. Based on chart reviews at two study sites, we estimate that 12 and 13% of inpatients during the baseline period will be referred for cessation counseling and will receive recommended pharmacotherapy, respectively. The projected study sample will have 81 and 83% power to detect a seven percentage-point difference in these process measures, respectively. These power calculations are based on a hierarchical linear model, with nurse treated as a random factor (and patient outcomes are clustered within nurse); intracluster correlation (ICC) was estimated to be 0.05 for assistance in quitting (which includes offering of pharmacotherapy and arranging follow-up), and 0.01 for abstinence, based on data from the AHRQ Smoking Cessation Guideline Evaluation Trial [77].

### Statistical analysis

The primary clinical endpoint of this analysis is seven-day PPA at three and six months. The primary process of care endpoints are referral of patients to telephone counseling (or other outpatient cessation counseling) and prescription of recommended pharmacotherapy for smoking cessation. To evaluate the relationship between the intervention and these outcomes, we will use hierarchical logistic regression to adjust for differences in potentially confounding patient and nurse characteristics between the baseline and intervention period cohorts. In these analyses, each patient will be nested within his/her admitting nurse, who will have primary responsibility for providing brief cessation counseling. To compare how the two arms differ in time to first relapse after hospital discharge, we will use Cox proportional-hazards survival analysis methods with time to relapse as the outcome [83]; in this analysis, intra-nurse correlation will be accounted for by using the robust sandwich estimator [84].

To evaluate the hypothesis that staff nurses will have improved decisional balance scores and higher ratings of self-efficacy in providing cessation counseling as a result of the intervention, we will compare values of these measures obtained at the end of the intervention period to pre-intervention values using a repeated measures ANOVA with site included as a covariate (or a site-stratified Wilcoxon signed-rank test, if the data are skewed or heavy-tailed). To determine whether the intervention effect (if any) is modified by nurses' perceptions of their work, we will check the interaction between treatment and nurse ratings of professional status and adequacy of staffing.

We will use an intent-to-treat approach in which patients are analyzed according to the period in which they were enrolled. In our analysis of three- and six-month cessation rates, those who are lost to follow-up will be considered failures (*i.e*., still smoking). Using this approach, however, some bias may be introduced on account of informative censoring (even if the pattern of missingness is evenly distributed across baseline and intervention periods). For this reason, we will also conduct analyses to examine the patterns of missingness, and will perform sensitivity analyses to compare intervention effects under a range of assumptions regarding the mechanism of missingness [85,86]. All tests will be two-sided and will use a p-value of 0.05 for statistical significance. To fit the hierarchical models, we will use SAS (PROC GLIMMIX).

### Approach to qualitative analysis

Interview and focus group transcripts will be imported into NVivo, a qualitative data management and analysis software package. Descriptive content analysis will be used to characterize inductively the issues raised by study participants [87,88]; this process will be performed separately for patients and nurses. Two reviewers, trained in qualitative research methods, will read the transcripts twice to identify pertinent issues and to construct a provisional coding structure before actual coding commences. The two reviewers will then code the transcripts by highlighting units of text that correspond to specific issues, including those reflected in the provisional coding structure. Inter-rater reliability for key issues will be determined. Similar issues will be grouped together under overarching domains. Domain characteristics, including universality (across all informants), idiosyncrasy (unique to one or two informants), and depth (types and richness of subcategories), will be assessed and descriptive statements about each domain will be developed using the patients' words. Reviewers will pay close attention to minority opinions that deviate from the dominant themes and emerging hypotheses [89].

### Cost effectiveness analysis

We will determine the cost-effectiveness ratio for the study intervention from the perspective of the VA health care system. We will account for cessation-related costs incurred during the initial hospitalization and six-month follow-up period in order to estimate the short-term incremental cost per self-reported quitter:



where cost_int _and cost_b _equal the average total direct VA resource cost for the intervention and the control groups, respectively, n_int _is the number of patients exposed to the intervention, and q_int _and q_b _are the six-month quit rates in the intervention and baseline periods, respectively. The denominator reflects change in the number of patients quitting as a result of the intervention. The numerator reflects the change in VA direct costs as a result of the intervention.

Costs related to implementation will include the following: time required for focus groups, initial training, and feedback sessions (both for project staff and nursing staff); nurse training materials, including internet tutorial; time required for CPRS programming to develop nursing reminders and prescription templates; and estimated time spent by peer leaders in coaching staff nurses. We will also include the salary of the full-time central research coordinator and the salary support for the external nurse facilitator, who will provide technical support for peer leaders and nursing staff at all sites. To calculate the costs related to delivery of smoking cessation services, we will estimate the time expended on bedside counseling of smokers by staff nurses, and then multiply this quantity by the average hourly salaries (including benefits) of VA nursing staff, based on VA Human Resources data. We will also track the time spent on telephone counseling, and estimate these costs by multiplying the hourly salary of a quit line counselor by the number of hours spent on telephone counseling. The costs of pharmacotherapy will be based on the quantity dispensed and the average wholesale price for each product (generic transdermal NRT, bupropion, and/or second-line drug therapies). We will also include the costs of patient education materials (brochures and educational video). For patients in each period, we will estimate the cost of outpatient visits for cessation counseling and/or smoking cessation pharmacotherapy (including management of adverse reactions) during six-month follow-up (by identifying cessation-related office visits and multiplying by the average cost for a physician visit).

To assess the robustness of the incremental cost per quitter to changes in effectiveness, we will perform one-way sensitivity analyses over plausible ranges of cost parameters and the six-month quit rate. The uncertainty of the incremental cost per quitter will also be estimated using non-parametric bootstrapping (with 1,000 replications), based on random sampling with replacement of a number of participants in the trial [90].

## Discussion

Although it is generally accepted that RCTs provide the highest level of evidence in health care research, randomization in the current study is neither feasible nor acceptable to study participants [24]. Moreover, RCTs are not always the best study design for evaluation of the implementation of complex interventions, which typically include multiple components, target multiple outcomes, or aim to achieve outcomes that are difficult to influence [91]. Using a quasi-experimental design, this study will fill an important gap in the evidence base for smoking cessation in hospitalized patients, as the effectiveness of brief bedside counseling combined with sustained relapse prevention is unclear. The two studies that combined brief inpatient counseling with sustained relapse prevention were limited by insufficient power [92], lack of data on the extent to which the relapse prevention component was implemented [92], and failure to include pharmacotherapy as part of intervention [93].

Limitations of the proposed study design deserve comment. Pre-post changes in outcome may be attributable to 'history' (the influence of events during the study that affect the study outcomes) or 'maturation' (the change of staff performance during the study related to the evolution of clinical skills) [25]. Potential Hawthorne effects should be minimized by employing a sufficiently long intervention period (six months). We will check for any time-related changes in cessation counseling or patients' intention to quit during the enrollment period in each hospital. This will allow us to closely monitor any secular trends (*e.g*., release of new smoking cessation aids, changes in hospital policies) that might impact upon delivery of cessation counseling or cessation outcomes. In addition, we will account for any confounder variables that show significant imbalances between baseline and intervention periods in multivariable analyses.

This project directly addresses one of the key recommendations to emerge from the VA Large Health Study: 'We must find ways to identify and target those ready to quit using tobacco so that limited resources have greater effect [2].' If the study intervention is shown to be effective, it will provide insights on how the CCM can be applied to promote implementation of smoking cessation guidelines in hospitals, and more generally, how to improve the continuity of preventive services as patients make the transition from the hospital to outpatient setting. The results of this research can be used by clinicians, quality managers, and VA decision makers to improve the quality of smoking cessation services (above and beyond 'ask' and 'advise' performance measures) [94,95]. If the study intervention leads to a 7% increase in quit rates, this would result in 7,000 new non-smokers annually if implemented system-wide (of the approximately 100,000 smokers who are admitted to VA hospitals annually).

### Ethics

This protocol was approved by the Institutional Review Board at the University of Iowa on 12 November 2008 (IRB protocol 200805711).

## Competing interests

The authors declare that they have no competing interests.

## Authors' contributions

DAK conceived the study, collected pilot data, and drafted the study protocol. MV, HR, SH, and MT helped to draft the study protocol. All authors provided critical review of the study protocol and approved the final manuscript.
